# Effectiveness of the Telemedical Lifestyle Intervention Program TeLIPro for Improvement of HbA_1c_ in Type 2 Diabetes: A Randomized-Controlled Trial in a Real-Life Setting

**DOI:** 10.3390/nu15183954

**Published:** 2023-09-12

**Authors:** Kerstin Kempf, Clara Dubois, Matthias Arnold, Volker Amelung, Nora Leppert, Sibel Altin, Markus Vomhof, Andrea Icks, Stephan Martin

**Affiliations:** 1 West-German Centre of Diabetes and Health, Düsseldorf Catholic Hospital Group, 40591 Düsseldorf, Germany; stephan.martin@uni-duesseldorf.de; 2 inav—Private Institute for Applied Health Services Research GmbH, 10117 Berlin, Germany; dubois@inav-berlin.de (C.D.); arnold@inav-berlin.de (M.A.); amelung@inav-berlin.de (V.A.); 3 German Institute for Telemedicine and Healthcare (DITG) GmbH, 40591 Düsseldorf, Germany; nora.leppert@gmail.com; 4 General Health Insurance Scheme (AOK Rheinland/Hamburg—Die Gesundheitskasse), 40213 Düsseldorf, Germany; sibelvildan.altin@rh.aok.de; 5 Institute for Health Services Research and Health Economics, German Diabetes Center, Leibniz Center for Diabetes Research at the Heinrich-Heine-University Düsseldorf, 40225 Düsseldorf, Germany; markus.vomhof@uni-duesseldorf.de (M.V.); andrea.icks@uni-duesseldorf.de (A.I.); 6 Institute for Health Services Research and Health Economics, Centre for Health and Society, Faculty of Medicine, Heinrich-Heine-University Düsseldorf, 40225 Düsseldorf, Germany; 7 German Center for Diabetes Research (DZD), 85764 Neuherberg, Germany; 8 Faculty of Medicine, Heinrich-Heine-University Düsseldorf, 40225 Düsseldorf, Germany

**Keywords:** type 2 diabetes, HbA_1c_, weight loss, cardiovascular risk factors, lifestyle intervention, health insurance

## Abstract

The effectiveness of the multimodal Telemedical Lifestyle Intervention Program (TeLIPro) was proven in the advanced stages of type 2 diabetes mellitus (T2DM). Since its therapeutic potential focusing on telemedical coaching without using a formula diet is unknown, we evaluated improvements in HbA_1c_, HbA_1c_ normalisation rate, cardiometabolic risk factors, quality-of-life, and eating behaviour in real life. In this randomized-controlled trial, AOK Rhineland/Hamburg insured T2DM patients (*n* = 1163) were randomized (1:1) into two parallel groups, and 817 received the allocated intervention. In addition to routine care, all participants got scales, step counters, and access to an online portal. The TeLIPro group additionally received equipment for self-monitoring of blood glucose and telemedical coaching. Data were collected at baseline, after 6 and 12 months of intervention as well as after a 6-month follow-up. The primary endpoint after 12 months was (i) the estimated treatment difference (ETD) in HbA_1c_ change and (ii) the HbA_1c_ normalisation rate in those with diabetes duration < 5 years. The TeLIPro group demonstrated significantly stronger improvements in HbA_1c_ (ETD −0.4% (−0.5; −0.2); *p* < 0.001), body weight, body-mass-index, quality-of-life, and eating behaviour, especially in T2DM patients with diabetes duration ≥ 5 years (ETD −0.5% (−0.7; −0.3); *p* < 0.001). The HbA_1c_ normalisation rate did not significantly differ between groups (25% vs. 18%). Continuous addition of TeLIPro to routine care is effective in improving HbA_1c_ and health-related lifestyle in T2DM patients with longer diabetes duration in real life.

## 1. Introduction

The increasing prevalence of type 2 diabetes mellitus (T2DM) is one major problem for the healthcare system. In Germany, at least 8 million people are currently affected. Based on health insurance data, it is estimated that this number will rise to 11.5 million T2DM patients by 2040 [1]. Diabetic complications exacerbate the problem, not only because of the health limitations of those affected but also because the emerging medical costs are an enormous burden for the healthcare system, e.g., for retinopathy (EUR 700), diabetic foot syndrome (EUR 1300), nephropathy (EUR 3400), myocardial infarction (EUR 8000–8700), stroke (EUR 10,000–11,200), amputation (EUR 14,300), and end-stage renal disease (EUR 22,700) in the quarter of event [2].

In Germany, diabetes care is guaranteed by approx. 300 clinics with diabetes departments, 1100 diabetes practices, and 60,000 general practitioners, with one general practitioner usually treating about 100 patients with T2DM. A second major problem is that due to the age structure, the number of general practitioners and diabetologists is declining, so there are already large regional differences in diabetes care, which will become even more pronounced in the coming years [3]. Medical innovations, such as telemedical coaching, are necessary to promote the self-empowerment of patients as additional offers to standard care and also to relieve physicians.

Since an unhealthy lifestyle is a leading cause of the development of T2DM, studies have shown that within the first years after diabetes diagnosis structured, non-pharmacological lifestyle interventions can significantly improve metabolic control [4,5,6], up to glycated haemoglobin (HbA_1c_) normalisation [7] and diabetes remission [8,9]. In this context, telemedical support programs were developed, which differed, for example, in the technical implementation, type of contact, or target parameters. In general, positive effects on diabetes control have been demonstrated [10,11,12,13,14]; however, scientifically evaluated, multimodal, structured lifestyle intervention programs that support diabetes care in a structured and comprehensive manner and that can be prescribed by general practitioners throughout Germany are missing. Therefore, the Innovation Funds of the German Federal Ministry of Health support medically innovative programs that aim to improve healthcare in Germany [15].

To close this supply gap, we previously developed the multimodal Telemedical Lifestyle Intervention Program (TeLiPro), a structured lifestyle intervention program combining telemonitoring of physical activity and weight, self-monitoring of blood glucose, telemedical coaching, an evaluated mental motivational training, and carbohydrate-reduced nutrition starting with a 12-week formula diet phase and evaluated the efficacy in a previous randomized-controlled trial (RCT) (NCT02066831) in poorly controlled T2DM [16]. Participants of the intervention group achieved a significantly higher HbA_1c_ reduction of −1.1 ± 1.2% (with an estimated treatment difference (ETD) of −0.8% (−1.1; −0.5); *p* < 0.0001) [16].

In order to test the TeLIPro telemedical coaching under real-life conditions, i.e., the setting in which TeLIPro would be offered if it would be included as an on-top treatment to routine care, the present RCT received a grant from the *Innovation Funds*. After a positive assessment by the *Joint Federal Committee* (Gemeinsamer Bundesausschuss, G-BA), TeLIPro might be prescribed by physicians and covered by health insurance in Germany.

Thus, the aim of the current study was to evaluate the potential impact of TeLIPro focusing on telemedical coaching without using a formula diet on metabolic control in real life. Therefore, we enrolled patients with T2DM of German statutory health insurance into an RCT. Based on our previous study in T2DM patients with longer-lasting (mean duration 11 years) and poorly controlled diabetes [16], we hypothesized that participation in TeLIPro would be associated with significantly stronger reductions in HbA_1c_, especially in those with a diabetes duration of ≥5 years. Since for patients with shorter diabetes duration, Lim et al. (<4 years) and Lean et al. (≤6 years) demonstrated a high potential for diabetes remission [8,9], the second hypothesis tested was that participation in TeLIPro would be associated with a higher HbA_1c_ normalisation rate after 12 months in patients with a diabetes duration of <5 years compared to control patients in routine care.

## 2. Materials and Methods

### 2.1. Study Design

The TeLIPro study is an RCT with two parallel groups analyzing the effect of telemedical coaching on HbA_1c_ in patients with T2DM. The first participant was enrolled on 25 September 2018; the last participant finished the follow-up on 21 September 2021. The study was conducted by the German Institute for Telemedicine and Healthcare in cooperation with the West-German Centre of Diabetes and Health in Düsseldorf, the General Health Insurance Scheme in North Rhine Westphalia and Hamburg (AOK Rheinland/Hamburg, Düsseldorf, Germany) based in Düsseldorf and evaluated by the private Institute for Applied Health Services Research (inav).

### 2.2. Study Population

Persons diagnosed with T2DM and insured by the AOK Rhineland/Hamburg were informed by their health insurance about the opportunity to participate in the study. Interested persons were eligible if they were 18–67 years old (for reflecting the working population), with an HbA_1c_ ≥ 6.5%, a body mass index (BMI) ≥ 27 kg/m^2^, internet access, and subscribed to the German Disease Management Program (DMP) Type 2 diabetes mellitus. Exclusion criteria were: chronic diseases other than T2DM and hypertension (e.g., cancer, dementia, psychoses, kidney insufficiency with glomerular filtration rate < 45 mL/min/1.73 m^2^, hearing loss), hospitalization during the last 3 months, acute chemotherapy, chronic cortisol treatment for longer than 6 weeks, pregnancy or breast-feeding, participation in another clinical trial during the last 6 months, or lack of knowledge of German.

### 2.3. Randomization and Masking

A serial study identification number (ID) was assigned by study staff to each participant who agreed to participate in the study, who had given written informed consent, and who fulfilled the inclusion and no exclusion criteria. The randomization list was electronically generated, and each ID was randomly assigned in a 1:1 ratio into one of the two parallel groups by a trial statistician. The trial statistician has no access to the list of IDs assigned to the names. The treating physicians and the outcome assessor were blinded for group assignment. For the participants and coaches, there was no blinding of assignment.

### 2.4. Procedures and Interventions

Participants of both the control and the TeLIPro group remained in routine care, i.e., quarterly DMP visits with their attending physician. Participants of both groups received self-monitoring telemedical devices, i.e., a scale (BF600, Beurer GmbH, Ulm, Germany; for weighing at least once/week), a step counter (AS 97, Beurer GmbH, Ulm, Germany; to be used on each day) and access to a secured online portal or App. Measured weight and steps were automatically transferred into the portal or App, and participants were able to enter additional health data. The TeLIPro group additionally received a blood glucose meter (Omnitest 5, B. Braun-Petzold GmbH, Melsungen, Germany) with test stripes for self-monitoring of blood glucose and according to participants’ individual needs 10–17 care calls (e.g., weekly in month 1, every second week in month 2–3, monthly in month 4–6, quarterly in month 7–12). Telemedical coaching was provided by diabetes assistants or diabetes consultants employed at the German Institute for Telemedicine and Healthcare, including technical advice and troubleshooting with telemedical devices, information about T2DM, healthy diet, and physical activity. For telemonitoring health data, the coaches also had access to the online portal or App. Weight development, step counts, and blood glucose profiles were discussed with regard to subjective obstacles or possibilities for lifestyle changes, and target agreements were made.

At baseline, after 6 and 12 months of intervention, as well as after 18 months (=6-month follow-up), health parameters (weight, BMI, blood pressure, and laboratory variables such as HbA_1c_, total cholesterol, high-density lipoprotein (HDL) cholesterol, low-density lipoprotein (LDL) cholesterol, and triglycerides) were provided by the attending physicians and local laboratories. By using push-up information and telephone calls, the participants were advised to load the health data into the online portal or App. Letters from the physicians, including the measured health data, were used for data completion and validation. Physical and mental wellbeing, impairment in quality of life and eating behaviour were assessed online using validated self-reporting questionnaires (i.e., the short form (SF) 12 questionnaire [17], the German version ‘Allgemeine Depressionsskala’ (ADS-L) of the Center for Epidemiological Studies Depression (CES-D) Scale [18], and the German version ‘Fragebogen zum Essverhalten’ (FEV) of the ‘Three-factor Eating Questionnaire’ [TFEQ] [19]. Regular data transfer (i.e., steps and body weight), logging into the online portal at least once a week, and answering the care calls were used as compliance measurements.

### 2.5. Outcomes

Based on our previous intervention in T2DM patients with longer-lasting and poorly controlled diabetes [16] and studies investigating HbA_1c_ normalisation and diabetes remission in patients with short-term diabetes duration [8,9], the primary endpoint was (i) the ETD of HbA_1c_ change after 12 months between groups, especially in patients with diabetes duration of ≥5 years and (ii) the HbA_1c_ normalisation rate after 12 months in T2DM patients with diabetes duration of <5 years compared between the TeLIPro and the control group. HbA_1c_ normalisation was defined as HbA_1c_ < 6.5% at the corresponding time point [7]. Diabetes duration was taken from the health insurance billing data and validated by the physician’s letter. Secondary endpoints were differences in responder rate (defined by a clinically relevant HbA_1c_ reduction of ≥0.5% [20]), body weight, BMI, cardiovascular risk factors, quality of life, and eating behaviour after 12 months as well as differences in all before mentioned parameters after 6 and 18 months.

### 2.6. Statistical Analysis and Power Calculation

Based on previous data of T2DM patients with longer-lasting and poorly controlled diabetes who participated in the TeLiPro telemedical lifestyle intervention program (including formula diet) and who achieved an HbA_1c_ reduction of 0.7 ± 1.3% [16], for the present study, an HbA_1c_ reduction of 0.5 ± 1.1% in the TeLIPro group and of 0.2 ± 0.9% in the control group after 12 months was assumed. The second calculation was based on previously obtained data for the TeLiPro application in another health insurance cohort of T2DM patients, who achieved an HbA_1c_ normalisation rate of 18% after 12 months of intervention [21]. Thus, for T2DM patients with a diabetes duration < 5 years, an HbA_1c_ normalisation rate of 18% was assumed for the TeLIPro group and 5% for the control group. To be still able to detect such differences with a power of 80% and a level of significance of 5% in the subgroups of patients with diabetes duration ≥ 5 and <5 years, respectively, at least 175 datasets (including HbA_1c_ values at baseline as well as after 12 months of intervention) in the control and in the TeLIPro group from patients with longer-lasting diabetes and 94 datasets per group from patients with shorter diabetes duration were required. Assuming a dropout rate of approximately 30%, a minimum of 768 participants should have been recruited.

Per-protocol analyses were performed. For analysis of inter-group differences (estimated treatment difference) in HbA1c and secondary endpoints, a linear mixed model analysis was used (R-package lme4 version 1.1.31 [22] and emmeans version 1.8.3 [23]) with a random effect for an identification number and fixed effects for the group, time point, group*time point. HbA1c and secondary endpoints were imputed using predictive mean matching (R-package mice version 3.15.0 [24]). BMI at baseline, HbA1c at baseline, age, sex, Elixhauser comorbidity index (=a measure of mortality risk used by health assurances which is based on the ICD10 classification [25]), and time point were used for prediction of missing values. Bonferroni–Holm correction was used to control for multiple testing. For analysis of inter-group difference relative risk (RR) in HbA_1c_ normalisation rate and clinically relevant HbA_1c_ reduction, logistic mixed model analysis was used. The level of significance (α) was 0.05. Data were analyzed using the statistical software *R* version 4.2.1.

## 3. Results

**Figure 1:**
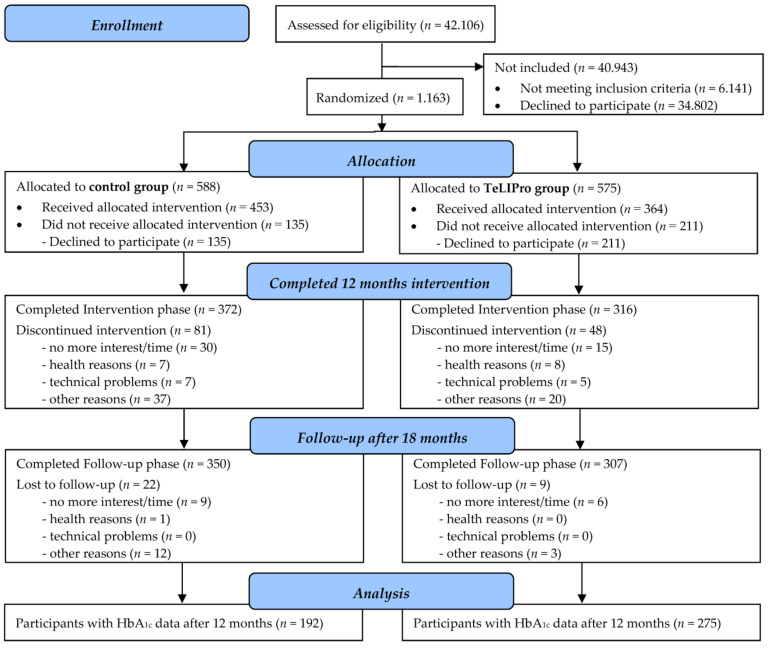
Flow chart.

A total of 1.163 patients were randomized into the control (*n* = 588) or the TeLIPro group (*n* = 575), of whom 453 and 364 (*n* = 817 in sum, set to 100%), respectively, received the allocated intervention. After 6 months, 751 participants (92%) remained in the study, i.e., 415 participants of the control group and 336 participants of the TeLIPro group. Moreover, 688 in sum (84%), i.e., 372 and 316 completed the 12 months of intervention (=final endpoint). Follow-up data were available for 657 (80%), i.e., 350 and 307 participants, respectively (Figure 1). Participants with data for HbA_1c_ after 12 months (*n* = 467 (57%); i.e., control group *n* = 192; TeLIPro group *n* = 275) were included in the analysis. No imported harms or unintended effects have been reported.

Baseline parameters did not differ significantly between groups with the exception of a lower baseline HbA_1c_ in the TeLIPro subgroup with diabetes duration ≥ 5 years and the overall study group compared to controls and a higher systolic blood pressure in the TeLIPro subgroup with diabetes durations ≥ 5 years (Table 1).

Moreover, baseline characteristics of completers and drop-outs did not significantly differ, except for group assignment, diabetes duration, and body weight, i.e., subjects in the control group (*p* = 0.039), with longer diabetes duration (*p* = 0.035) and higher body weight (*p* = 0.027) were more likely to drop out (Table 2).

**Figure 2:**
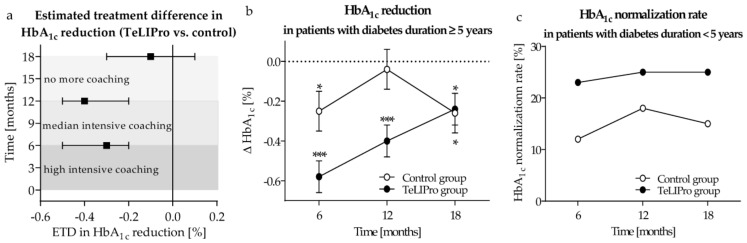
Reduction of HbA_1c_ and HbA_1c_ normalisation rate. (**a**) While participants of the control group remained in routine care, the TeLIPro group obtained highly intensive coaching during months 1–6, with median intensive coaching in months 7–12 followed by a 6-month phase without coaching. The estimated treatment difference (ETD) (95% confidence interval) in HbA_1c_ reduction was estimated after 6, 12, and 18 months. Analyses were performed using a linear mixed model without adjustments. (**b**) In patients with diabetes duration ≥ 5 years, HbA_1c_ was measured after 6 and 12 months of intervention as well as after 6 months of follow-up. Differences to baseline were estimated for the TeLIPro (*n* = 165; black circles) and the control group (*n* = 115; white circles) using a linear mixed model (* *p* < 0.05; *** *p* < 0.001). Shown are means ± standard error of the mean. (**c**) HbA_1c_ normalisation was defined as HcA1c < 6.5%. HbA_1c_ normalisation rates in patients with diabetes duration < 5 years were compared in the TeLIPro (*n* = 110; black circles) vs. the control group (*n* = 77; white circles) using a logistic mixed model.

A treatment superiority was observed for the TeLIPro group with an ETD in HbA_1c_ reduction of −0.3% [−0.5; −0.2] (*p* < 0.01) during the 6 months of high intensive coaching and −0.4% [−0.5; −0.2] (*p* < 0.001) after 12 months of intervention. Six months after the end of coaching, ETD did not remain statistically significant (Figure 2a).

While in the control group of patients with diabetes duration ≥ 5 years there was no significant improvement of HbA_1c_ after 12 months, the TeLIPro group demonstrated a mean HbA_1c_ reduction of −0.4 ± 0.1% (*p* < 0.001) (Figure 2b). Moreover, 6 months after the end of the intervention, HbA_1c_ values remained significantly reduced compared to baseline, although the reduction was comparable in both groups (−0.3 ± 0.1% in control vs. −0.2 ± 0.1% in the TeLIPro group). Thus, the ETD was −0.4% (−0.6; −0.2) (*p* < 0.01) after 6 months and −0.5% (−0.7; −0.3) (*p* < 0.001) after 12 months. There was no significant treatment difference after the follow-up phase. Sensitivity analyses with adjustment for sex, age, Elixhauser comorbidity index, BMI at baseline, and HbA_1c_ at baseline confirmed these results (Table 3).

Although not reaching a statistically significant difference, the HbA_1c_ normalisation rate in the subgroup with diabetes duration < 5 years tended to be higher in the TeLIPro group (*n* = 27; 25%) compared to the control group (*n* = 14; 18%) during the 12-month intervention phase. Achieved HbA_1c_ normalisation rates could be maintained during the 6-month follow-up phase (Figure 2c).

The responder analysis showed that the RR for a clinically relevant reduction in HbA1c of at least 0.5% was significantly higher in the TeLIPro group after 12 months (RR 1.9 [1.3; 3.0], *p* = 0.002) respectively (Table 3). This difference in RR in favour of the TeLIPro group could also be found in those with diabetes duration < 5 years after 12 months (RR 2.1 [1.3; 3.0]; *p* = 0.004).

Although there was no significant difference in RR of achieving HbA_1c_ normalisation for patients in the TeLiPro vs. the control group in those with shorter diabetes duration (Table 3), significantly higher RR for reaching HbA_1c_ normalisation were observed in the complete TeLIPro group as well as in those with longer diabetes duration at all time points.

Moreover, significant treatment priority for body weight, BMI, physical and mental wellbeing, impairment of quality of life, and eating behaviour was observed for the TeLIPro group compared to the control group after 12 months of intervention (Table 4). With an exception for physical and mental wellbeing, all effects remained statistically significant after Bonferroni correction for multiple testing. However, they did not remain significant after the end of coaching (=6-month follow-up phase).

## 4. Discussion

The TeLIPro RCT proved that in a real-life setting, T2DM patients with longer diabetes duration can significantly benefit from the evaluated telemedicine-based lifestyle intervention. After 12 months of intervention, patients in the TeLIPro group achieved a significantly greater reduction in HbA_1c_, body weight, and BMI compared to controls who remained in routine medical care without additional telemedical coaching. Moreover, the TeLIPro group reported significant improvements in quality of life and eating behaviour. However, the missing effects in the follow-up phase show that coaching must be offered continuously in order to maintain these effects.

The current TeLIPro study in health insurance patients with T2DM is the follow-up and real-life verification study of the TeLiPro study in T2DM patients with insufficient metabolic control [16]. This previous trial combined five lifestyle intervention components, i.e., telemonitoring of physical activity and weight, self-monitoring of blood glucose, telemedical coaching, medical–mental motivation, and nutritional advice starting with an intensive meal-replacement phase [16]. After 3 months of intervention and 9 months of follow-up, the observed HbA_1c_ reduction of −1.1 ± 1.2% had been significantly higher in the intervention group vs. −0.2 ± 0.8% in the control group with an ETD of −0.8% (−1.1; −0.5) (*p* < 0.0001). In the present study, the participants in the TeLIPro group were offered the same intervention components except for the formula diet. The effects in the current study with an ETD of −0.5% [−0.7; −0.3] (*p* < 0.001) are comparable but somewhat lower.

A possible explanation for this might be that the first study was advertised via general practitioners and newspaper articles, and the participants had registered of their own accord. In the later study, participation was offered to patients with T2DM by their health insurance; thus, patients with little motivation and less drive might have been included. But this represents the situation in real life. Nevertheless, after 12 months of intervention, 688 persons (84%) regularly transferred their steps and body weight, logged into the online portal at least once a week, and answered the care calls, though they were rated to be compliant. Therefore, the lack of meal replacement is seen as the main reason for the lower effects on HbA_1c_. During the telephone coaching, the participants were given nutritional advice with a focus on carbohydrate reduction and an increase in unsaturated lipid intake. These dietary interventions have the potential to prevent the development of T2DM [26,27] cardiovascular endpoints [28] and are associated with reduced mortality [29]. However, in contrast to the previous study, the intervention was not started with a formula diet for meal replacement, which apparently influenced the long-term HbA_1c_ reduction. In previous studies, we could show that starting with an intensive meal replacement supported maintaining the achieved HbA_1c_ reduction in the long term [30]. When the real-life verification study had been designed, the use of formula diets as a treatment option for T2DM was still rejected by diabetes associations and not supported by health insurance. In the meantime, however, the beneficial effects of formula diets have been documented in a large number of studies in different countries, with a substantial potential for diabetes remission [8,9,31,32,33], which is defined as HbA_1c_ < 6.5% measured at least three months after termination of glucose-lowering pharmacotherapy [34]. Therefore, meal-replacement therapy has become included in the guidelines of the national and international diabetes associations as a baseline therapy option for the treatment of T2DM [35,36].

Standard care with basic diabetes education was assumed to enable patients to carry out a successful lifestyle intervention on their own; however, a variety of studies rejected this postulation [37]. Intensive accompaniment during the intervention with strict carbohydrate reduction, quick reduction in insulin levels, and high weight reduction increases the chance of success [38]. Telemedical coaching is particularly important in this context to guarantee that participants understand the reasons why they should change their diet and lifestyle [12] because studies have shown that telephone calls alone are not sufficient to alter behaviour [39]. Strengthened cognitive control, reduced suggestibility, fewer feelings of hunger, and increased quality of life [16,40] underline the alterations in eating behaviour and lifestyle. Nevertheless, weight and HbA_1c_ values often re-increase after the end of intervention [41,42]. Based on these observations, many lifestyle interventions were dismissed as not sustainable, especially for the reduction of all-cause mortality [43]. In the meantime, however, the potential of lifestyle interventions has been recognized so that they have even been included as basic therapy in the current diabetes guidelines [35,36]. However, the same demands would never have been placed on pharmaceutical diabetes therapy. They are understood to be life-long. During the 3-year follow-up of the original TeLiPro study, we were able to show that the re-increase in HBA_1c_ after the end of the intervention could be stopped and Hba_1c_ could be reduced again if the nutritional intervention and telemedical coaching were resumed [44]. Therefore, a rethinking of the sustainability of telemedically supported lifestyle interventions should be carried out, and they might be used as continuous on-top therapy options in addition to routine care. In this context, cost-benefit calculations are necessary.

However, in the meantime, health devices with transfer functions have become state-of-the-art and are no longer a cost driver. Moreover, the open system architecture of the portal ensures interoperability. As an end-to-end solution, the online portal supports the elimination of fragmentation in healthcare. By integrating the numerous devices available on the market, patients can use their own and already available devices in the future. Personnel-intensive delivery methods are successful, and new communication approaches via apps show impressive results [45,46]. Further analyses should investigate economic aspects and the impact on the reduction of cardiometabolic endpoints.

This study has several strengths and limitations that need to be mentioned. More than 42.000 health-insured persons have been invited for participation in the study, of whom only 1.163 were interested. There was a relatively high number of persons who did not receive the allocated intervention. When they had been invited by their health insurance to participate in the study, they agreed in the first step but then withdrew consent after randomization. This is a common problem in real-life health insurance studies and might be overcome by performing the randomization in a later phase, e.g., after the first log-in to the online portal. However, the drop-out rate for the whole study population that received the allocated intervention was only 16% during the 12-month study period and a further 4% in the 6-month follow-up phase, largely without significant differences between completers and dropouts at baseline. It could be hypothesized that this randomization procedure might have led to a selection bias since the control group and the TeLIPro group differ in baseline HbA_1c_. However, since the baseline HbA_1c_ in the control group was higher, and studies have shown that a higher baseline Hba_1c_ facilitates the Hba_1c_ reduction [47], the observed treatment superiority of the TeLIPro group was rather underestimated.

An important strength and, at the same time, a limitation was the real-life study design, leaving patients in routine care at their general practitioner or diabetologist with the intervention as an on-top supply. A disadvantage and possibly the main limitation of the study is that due to the study design with the participants entering the data by themselves into the portal, the data sets were partly incomplete (also for HbA_1c_), and only the data sets containing HbA_1c_ at baseline and after 12 months were evaluated. For quality control, the entered data could be validated by the study staff using the physician’s letters. One aspect of the real-world approach was to enhance the self-empowerment and personal responsibility of participants, not only in lifestyle change but also in self-monitoring of health parameters. This included the data input into the online portal. Although 84% received the allocated intervention until month 12, only 57% (i.e., 42% of the control and 76% of the TeLIPro group) managed to enter their HbA_1c_ values into the online portal. Thus, the rate of missing HbA_1c_ values in the control group was nearly twice that in the intervention group. This phenomenon has previously been observed [16] with less compliance in the control group with routine care. In order to overcome this problem, the control group was also equipped with telemedical devices; however, the pure self-monitoring opportunity was not enough to keep up the motivation. Ultimately, this once again illustrates the immense bond and commitment that arises from telemedical coaching.

It also might be criticized that the clinical variables had been measured in local laboratories. Nevertheless, since laboratory measurements were consistently performed at the same laboratory and were reported in written form by the attending physician using the standardized DMP documentation, intra-individual differences should not have been affected. Moreover, we observed a very high HbA_1c_ normalisation rate in the control group, which was not significantly different from those in the TeLIPro group but higher than in comparable trials focusing on diabetes remission [8,9]. This could certainly be partially explained by the support provided by self-monitoring devices and portal usage. However, the most obvious explanation for this might be the fact that due to the study design, HbA_1c_ normalisation was just defined as HbA_1c_ < 6.5% without the requirement that the antidiabetic pharmacotherapy must have been discontinued for at least three months. If you use this definition, the results are comparable to other studies that use the same definition for HbA_1c_ normalisation [7]. Therefore, the observed HbA_1c_ normalisation rates mainly represent the well-adjustable metabolic control in early diabetes stages.

## 5. Conclusions

The study demonstrated that the TeLIPro lifestyle intervention can significantly improve HbA_1c_ levels and is an effective on-top tool for T2DM patients with longer-lasting diabetes in addition to routine care. For maintenance of the improvements, coaching should be offered continuously. After this confirmatory approach, TeLIPro might be provided nationwide by health assurances.

## Figures and Tables

**Table 1:** Baseline characteristics.

	Control Group			TeLIPro Group		
Parameters	All (*n* = 453)	Diabetes Duration < 5 Years (*n* = 182)	Diabetes Duration ≥ 5 Years (*n* = 271)	All (*n* = 364)	Diabetes Duration < 5 Years (*n* = 143)	Diabetes Duration ≥ 5 Years (*n* = 221)
Sex male/female (%)	65/35	65/35	65/35	62/38	62/38	62/38
Age (years)	54 ± 9	51 ± 10	56 ± 8	55 ± 9	52 ± 10	57 ± 8
HbA_1c_ (%)	7.8 ± 1.3	7.6 ± 1.4	8.0 ± 1.3	7.6 ± 1.2 *	7.5 ± 1.1	7.7 ± 1.2 **
Body weight (kg)	103 ± 22	106 ± 25	102 ± 19	104 ± 23	108 ± 24	102 ± 22
BMI (kg/m^2^)	34.4 ± 6.5	35.1 ± 7.2	33.9 ± 6.1	34.8 ± 7.0	35.7 ± 7.8	34.2 ± 6.4
Systolic BP (mmHg)	133 ± 16	133 ± 15	133 ± 16	134 ± 17	132 ± 20	135 ± 14 *
Diastolic BP (mmHg)	82 ± 9	82 ± 9	81 ± 9	82 ± 11	81 ± 11	82 ± 11
Total cholesterol (mg/dL)	194 ± 48	197 ± 46	193 ± 49	195 ± 40	200 ± 38	193 ± 42
HDL cholesterol (mg/dL)	47 ± 18	47 ± 19	47 ± 18	44 ± 15	42 ± 11	46 ± 17
LDL cholesterol (mg/dL)	123 ± 39	128 ± 41	120 ± 38	123 ± 36	128 ± 33	120 ± 38
Triglycerides (mg/dL)	211 ± 128	208 ± 117	212 ± 135	208 ± 121	202 ± 106	211 ± 129
Physical wellbeing (au) ^a^	41.0 ± 10.2	42.2 ± 10.5	40.2 ± 9.9	41.2 ± 10.2	41.1 ± 10.1	41.2 ± 10.4
Mental wellbeing (au) ^a^	48.4 ± 11.2	47.7 ± 11.4	48.8 ± 11.0	48.8 ± 10.7	47.9 ± 10.2	49.4 ± 11.0
Impairment of QoL (au) ^b^	14.4 ± 9.8	15.3 ± 10.3	13.9 ± 9.4	14.5 ± 9.4	14.9 ± 9.0	14.2 ± 9.6
Cognitive control (au) ^c^	7.5 ± 4.4	7.4 ± 4.4	7.6 ± 4.3	7.5 ± 4.2	7.5 ± 4.3	7.5 ± 4.1
Suggestibility (au) ^c^	6.2 ± 3.5	6.0 ± 3.2	6.4 ± 3.6	6.2 ± 3.4	6.1 ± 3.4	6.2 ± 3.4
Hunger (au) ^c^	5.5 ± 3.6	5.3 ± 3.4	5.6 ± 3.8	5.7 ± 3.7	5.5 ± 3.8	5.4 ± 3.7

Data are *n* (%) or mean ± SD. Data were missing for body weight (*n* = 37; 5%), BMI (*n* = 37; 5%), systolic (*n* = 125; 15%) and diastolic blood pressure (BP; *n* = 135; 17%), total (*n* = 206; 25%), high-density lipoprotein (HDL; *n* = 299; 37%), and low-density lipoprotein (LDL) cholesterol (*n* = 121; 15%), and triglycerides (*n* = 256; 31%). Between-group differences (TeLIPro vs. control) were analyzed by the Mann–Whitney U test or Chi^2^ test (* *p* < 0.05; ** *p* < 0.01); ^a^ Determined using SF-12 [17]; ^b^ German version of the ‘General Depression Scale’ (CES-D) [18]; ^c^ German version of the ‘Three-factor Eating Questionnaire’ (TFEQ) [19]; QoL, quality of life; au, arbitrary units.

**Table 2:** Baseline characteristics of completers and dropouts.

Parameters	Completers (*n* = 657)	Drop Outs (*n* = 160)
Control group/TeLiPro group (%)	53/47	64/36 *
Diabetes duration < 5/≥5 years (%)	41/59	34/66 *
Sex male/female (%)	64/36	63/37
Age (years)	54 ± 9	55 ± 9
HbA_1c_ (%)	7.7 ± 1.3	7.7 ± 1.3
Body weight (kg)	104 ± 23	100 ± 20 *
BMI (kg/m^2^)	34.8 ± 7.0	33.5 ± 6.5
Systolic BP (mmHg)	134 ± 17	133 ± 14
Diastolic BP (mmHg)	82 ± 10	81 ± 8
Total cholesterol (mg/dL)	194 ± 46	198 ± 38
HDL cholesterol (mg/dL)	46 ± 16	46 ± 19
LDL cholesterol (mg/dL)	123 ± 39	124 ± 35
Triglycerides (mg/dL)	210 ± 129	204 ± 105
Physical wellbeing (au)	41.1 ± 10.3	40.9 ± 9.7
Mental wellbeing (au)	48.8 ± 11.0	47.7 ± 10.9
Impairment of QoL (au)	14.3 ± 9.5	15.0 ± 9.9
Cognitive control (au)	7.6 ± 4.3	7.4 ± 4.3
Suggestibility (au)	6.2 ± 3.5	6.2 ± 3.3
Hunger (au)	5.5 ± 3.7	5.4 ± 3.7

Data are *n* (%) or mean ± SD. Participants who completed the 12 months of intervention as well as the 6 months of follow-up were defined as completers. Between-group differences were analyzed by the Mann–Whitney U test or Chi^2^ test (* *p* < 0.05). For further details, please see legend in Table 1.

**Table 3:** ETD of HbA_1c_ reduction with sensitivity analysis, relative risk for HbA_1c_ normalisation, and clinically relevant HbA_1c_ reduction.

Time (Months)	6	12	18
Estimated treatment difference (ETD)			
ETD (TeLIPro vs. control)	−0.3 (−0.5; −0.2) **	−0.4 (−0.5; −0.2) ***	−0.1 (−0.3; 0.1)
Model 2	−0.3 (−0.5; −0.1) **	−0.3 (−0.5; −0.1) **	0.1 (−0.1; 0.3)
Model 3	−0.3 (−0.5; −0.2) **	−0.4 (−0.6; −0.2) ***	−0.0 (−0.2; 0.2)
ETD (diabetes duration ≥ 5 years)	−0.4 (−0.6; −0.2) **	−0.5 (−0.7; −0.3) ***	−0.2 (−0.4; 0.0)
Model 2	−0.4 (−0.6; −0.1) **	−0.4 (−0.6; −0.2) **	−0.1 (−0.2; 0.3)
Model 3	−0.5 (−0.7; −0.3) ***	−0.5 (−0.7; −0.3) ***	−0.1 (−0.3; 0.2)
ETD (diabetes duration < 5 years)	−0.2 (−0.4; 0.1)	−0.2 (−0.5; 0.1)	0.0 (−0.3; 0.4)
Model 2	−0.1 (−0.5; 0.2)	−0.2 (−0.5; 0.1)	0.1 (−0.3; 0.5)
Model 3	−0.1 (−0.4; 0.2)	−0.2 (−0.5; 0.1)	0.1 (−0.2; 0.5)
Responder analysis (relative risk (RR) for clinically relevant HbA_1c_ reduction)			
RR (TeLIPro vs. control)	1.6 (1.1; 2.0) *	1.9 (1.3; 3.0) **	1.4 (0.9; 2.0)
RR (diabetes duration ≥ 5 years)	1.8 (1.0; 3.0)	1.7 (0.9; 3.0)	1.4 (0.7; 3.0)
RR (diabetes duration < 5 years)	1.4 (0.9; 2.0)	2.1 (1.3; 4.0) **	1.4 (0.8; 3.0)
HbA_1c_ normalisation			
RR (TeLIPro vs. control)	3.4 (1.7; 6.9) ***	2.1 (1.1; 3.9) *	2.8 (1.3; 6.1) **
RR (diabetes duration ≥ 5 years)	6.3 (2.9; 18.5) ***	3.1 (1.1; 8.6) *	5.4 (1.5; 19.3) **
RR (diabetes duration < 5 years)	2.0 (0.8; 4.8)	1.4 (0.6; 3.1)	1.7 (0.6; 4.7)

Shown are model-based estimators and 95% confidence intervals of the ETD in HbA_1c_ reduction. The analyses included all datasets with an HbA_1c_ value at baseline and after 12 months (TeLIPro *n* = 275 (subgroup with diabetes duration ≥ 5 years *n* = 165; with diabetes duration < 5 years *n* = 110); control *n* = 192 (diabetes duration ≥ 5 years *n* = 115; <5 years *n* = 77). Missing values after 18 months (*n* = 122; 26%) were imputed using predictive mean matching. Analyses were performed using linear mixed models including all available values at the corresponding time point: in the basic mixed model without adjustments; model 2 = basic mixed model + adjustment for sex, age, Elixhauser comorbidity index, BMI at baseline; model 3 = model 2 + adjustment for HbA_1c_ at baseline Responder have been defined by a clinically relevant HbA_1c_ reduction of at least 0.5%. HbA_1c_ normalisation was defined as HbA_1c_ < 6.5%. RR and 95% confidence intervals were estimated using a logistic mixed model (* *p* < 0.05; ** *p* < 0.01; *** *p* < 0.001).

**Table 4:** Estimated treatment differences of secondary endpoints after 12 months.

Parameters	Control Group (*n* = 192)	TeLIPro Group (*n* = 275)	ETD (TeLIPro vs. Control)	*p*
HbA_1c_ (%)	−0.0 ± 0.1	–0.4 ± 0.1	−0.4 (−0.5; −0.2)	**<0.001**
Body weight (kg)	–0.7 ± 0.4	–2.9 ± 0.3	−2.2 (−1.3; −3.0)	**<0.001**
BMI (kg/m^2^)	–0.2 ± 0.1	–0.9 ± 0.1	−0.8 (−0.5; −1.0)	**<0.001**
Systolic BP (mmHg)	–0.3 ± 1.1	–1.5 ± 0.9	−1.2 (1.5; −3.9)	0.384
Diastolic BP (mmHg)	–0.8 ± 0.9	–1.1 ± 0.7	−0.3 (1.7; −2.3)	0.770
Total cholesterol (mg/dL)	–5.3 ± 2.4	–6.6 ± 2.5	−1.3 (4.8; −7.5)	0.672
HDL cholesterol (mg/dL)	0.8 ± 1.5	1.4 ± 1.2	0.6 (3.3; −2.2)	0.675
LDL cholesterol (mg/dL)	–4.9 ± 3.5	−5.7 ± 2.9	−0.8 (4.9; −6.5)	0.787
Triglycerides (mg/dL)	–12.8 ± 8.3	–14.1 ± 9.3	−1.3 (16.6; −19.1)	0.890
Physical wellbeing (au)	1.4 ± 0.5	2.9 ± 0.4	1.4 (2.7; 0.2)	0.023
Mental wellbeing (au)	–1.6 ± 0.7	0.3 ± 0.5	1.9 (3.6; 0.2)	0.029
Impairment of QoL (au)	1.1 ± 0.9	–1.3 ± 0.5	−2.3 (−0.9; −3.7)	**0.001**
Cognitive control (au)	0.9 ± 0.3	2.7 ± 0.2	1.8 (2.4; 1.1)	**<0.001**
Suggestibility (au)	–0.5 ± 0.2	–1.1 ± 0.1	−0.6 (−0.2; −1.0)	**0.003**
Hunger (au)	–0.7 ± 0.2	–1.8 ± 0.2	−1.1 (−0.6; −1.5)	**<0.001**

Shown are within-group differences (12 months vs. baseline) ± SEM and the ETD (95% confidence interval). All datasets with an HbA_1c_ value after 12 months were included. Data were missing for body weight (*n* = 34; 7%), BMI (*n* = 34; 7%), systolic (*n* = 124; 27%) and diastolic blood pressure (BP; *n* = 127; 27%), total (*n* = 196; 42%), HDL (*n* = 241; 52%), and LDL cholesterol (*n* = 116; 25%), triglycerides (*n* = 217; 46%), physical (*n* = 14; 3%) and mental wellbeing (*n* = 14; 3%), impairment of quality of life (*n* = 18; 4%), cognitive control of eating behaviour (*n* = 22; 4%), suggestibility (*n* = 22; 4%), and hunger (*n* = 22; 4%). Missing data were estimated using predictive mean matching. Analyses were performed using a linear mixed model without adjustment. Values with significant difference after Bonferroni correction were written in bold. For further details, please see legend in Table 1.

## Data Availability

The data that support the findings of this study are not openly available due to reasons of sensitivity and are available from the corresponding author upon reasonable request.
